# Supplementary material for: Complexome profiling on the Chlamydomonas lpa2 mutant reveals insights into PSII biogenesis and new PSII associated proteins
Source: J Exp Bot. 2021 Aug 26;73(1):245–62. doi: 10.1093/jxb/erab390 (PMC8730698; doi:10.1093/jxb/erab390)
Supplement: erab390_suppl_Supplementary_Dataset_S1 [file erab390_suppl_supplementary_dataset_s1.zip › Supplemental Dataset 1 - Excel List and all profiles/plots/CPLD55_Cre13.g585301.html]

Trivial name: CPLD55  
  
Euclidean distance: 11123.07  
Mean Intensity (WT): 670.85  
Mean Intensity (Mut): 361.87  
Distance: 16.58  
  
MapMan: lipid metabolism.glycolipid synthesis.MGDG synthase  
  
p value of intensity sums Welch test: 0.3981
